# Green cardamom supplementation improves serum irisin, glucose indices, and lipid profiles in overweight or obese non-alcoholic fatty liver disease patients: a double-blind randomized placebo-controlled clinical trial

**DOI:** 10.1186/s12906-019-2465-0

**Published:** 2019-03-12

**Authors:** Milad Daneshi-Maskooni, Seyed Ali Keshavarz, Mostafa Qorbani, Siavash Mansouri, Seyed Moayed Alavian, Mahtab Badri-Fariman, Seyed Ali Jazayeri-Tehrani, Gity Sotoudeh

**Affiliations:** 1 Department of Community Nutrition, School of Nutritional Sciences and Dietetics, Tehran University of Medical Sciences, Tehran, Iran; 2 School of Medicine, Jiroft University of Medical Sciences, Jiroft, Kerman Iran; 3 Department of Clinical Nutrition, School of Nutritional Sciences and Dietetics, Tehran University of Medical Sciences, Tehran, Iran; 4 Non-Communicable Diseases Research Center, Alborz University of Medical Sciences, Karaj, Iran; 5 Gastroenterohepatology Department, National Iranian Oil Company (NIOC) Central Hospital, Tehran, Iran; 6 Baqiyatallah Research Center for Gastroenterology and Liver Diseases (BRCGL), Baqiyatallah University of Medical Sciences, Tehran, Iran

**Keywords:** Non-alcoholic fatty liver disease, Green cardamom, Overweight or obesity, Irisin, Glucose indices, Lipids

## Abstract

**Background:**

Despite the reported health effects of cardamom on dyslipidemia, hepatomegaly, and fasting hyperglycemia, no human research has studied its potency in non-alcoholic fatty liver disease (NAFLD) as the hepatic part of metabolic syndrome. Our aim was determining the effects of green cardamom (GC) on serum glucose indices, lipids, and irisin level among overweight or obese NAFLD patients.

**Methods:**

The place of participant recruitment was the polyclinic of the National Iranian Oil Company (NIOC) central hospital in Tehran. Based on the ultrasonography and eligibility criteria, 87 participants were randomly divided into two groups as cardamom (*n* = 43) or placebo (*n* = 44). The supplementation was two 500 mg capsules 3 times/day with meals for 3 months. Serum irisin, fasting blood sugar (FBS), insulin (FBI), total cholesterol (TC), triglyceride (TG), low-density lipoprotein cholesterol (LDL-c), and high-density lipoprotein cholesterol (HDL-c) were measured. Quantitative insulin sensitivity check index (QUICKI) and homeostasis model assessment-insulin resistance (HOMA-IR) were also calculated.

**Results:**

In comparison with placebo, GC significantly increased irisin, HDL-c, and QUICKI and decreased FBI, TG, LDL-c, HOMA-IR, and the grade of fatty liver (*P* < 0.05). After adjustment for confounders, the changes were similar (*P* < 0.05) with an exception for LDL-c which had a trend (*P* = 0.07). The differences in FBS, TC, and body mass index (BMI) were not significant (*P* > 0.05).

**Conclusion:**

GC supplement improved the grade of fatty liver, serum glucose indices, lipids, and irisin level among overweight or obese NAFLD patients. The changes in these biomarkers may yield beneficial effects on NAFLD. Further trials on the efficacy of GC for clinical practice are suggested.

**Trial registration:**

Iranian Registry of Clinical Trials, IRCT2015121317254N4. Registered 27/12/2015,

**Electronic supplementary material:**

The online version of this article (10.1186/s12906-019-2465-0) contains supplementary material, which is available to authorized users.

## Background

Non-alcoholic fatty liver disease (NAFLD), as the most common liver disease [1, 2], occurs when fat is deposited (steatosis) in the liver without excessive alcohol use. The most extreme NAFLD form is non-alcoholic steatohepatitis (NASH) [3]. NAFLD is related to insulin resistance and metabolic syndrome (obesity, combined hyperlipidemia, diabetes mellitus type 2, and high blood pressure). It may respond to treatments originally developed for other insulin-resistant conditions such as type 2 diabetes mellitus [3, 4].

The global prevalence of NAFLD is emerging as 25.2% [5]. However, up to 80% of obese people have NAFLD [6–8]. The usual risk factors for NAFLD are obesity, impaired blood glucose regulation [9, 10], dyslipidemia, and older age [11, 12]. Inflammation and oxidative stress are the main inducers of insulin resistance that influence the pathology of NAFLD [13, 14]. Insulin resistance has a direct association with liver fat content [15].

Irisin, as a myokine [16] and adipokine [17], has a direct relationship with exercise and is related inversely to the triglyceride content of hepatocytes [16]. Irisin can modify insulin sensitivity [16, 18–21] by modulating glucose and lipid metabolism [19, 22] and enhancing uncoupling protein-1 (UCP1) expression which stimulates thermogenesis [19, 20, 23]. Regular exercise training and improving lifestyle [18, 20, 21, 23, 24] improve secretion of irisin by increasing peroxisome proliferator-activated receptor gamma (PPAR-γ) coactivator 1-alpha (PGC-1α). PGC-1α is a transcriptional coactivator which regulates the genes involved in energy metabolism through interacting with the PPAR-γ [25]., According to a study in obese NAFLD patients, liver enzymes and triglyceride contents had an inverse relationship with serum irisin [26]. Thus, irisin may play an important role in the amelioration of hepatic diseases especially NAFLD [16].

The dietary polyphenols as anti-oxidant and anti-inflammatory compounds have important roles [27]. GC (*Elettaria cardamomum*) from the ginger family as “the queen of spices” consists of numerous polyphenols such as quercetin [28], which suppress nuclear factor-kappa B (NF-κB) [28–31]. This factor is a protein complex involved in the control of cytokine production and cellular responses to stimuli such as inflammatory cytokines and oxidative stress [32]. Some of the polyphenols such as quercetin and resveratrol can activate PGC-1α [33, 34]. According to a study on adipocytes, quercetin increased the gene expression of irisin [17]. So, GC may influence serum irisin, insulin resistance, and hepatic steatosis [16].

Weight loss and physical activity are the common therapeutic approaches of NAFLD [35, 36]. Due to the disputable treatment of NAFLD, new therapies may be necessary for the amelioration of NAFLD [37]. Because of the challenge of weight loss and physical activity for a long time [38], changing the dietary ingredients may be an effective approach [39–41]. Important and different health effects have been reported for GC including antioxidant and anti-inflammatory effects [42–44]. 1,8-cineole and alpha-terpinyl acetate are the two major components of cardamom volatile oil [45].

Cardamom in many animal models has improved glucose metabolism [46–48]. Nevertheless, similar human studies are very limited. In Only two distinct studies of GC supplementation, changes of FBS and insulin sensitivity in overweight or obese pre-diabetic women [49, 50] and glycemic indices (FBS, insulin, and HbA1c) among type 2 diabetic patients [51], were not significant. However, insulin sensitivity in pre-diabetic women revealed a significant improvement across the cardamom group [49].

Due to the important effects of irisin in various metabolic pathways, its hepatic role should be studied. The stimulation of irisin secretion by GC in overweight or obese NAFLD patients should also be assessed. Meanwhile, the GC effects on serum irisin levels, glucose indices, and lipids in overweight or obese NAFLD patients have not previously been investigated. So, this study was planned to determine the efficacy of GC on serum irisin levels, glucose profiles, and lipids in these patients.

## Methods

### Study design and subjects

The ethics committee of Tehran University of Medical Sciences approved this double-blind randomized placebo-controlled clinical trial under the code of IR.TUMS.REC.1394.791. The study was registered to Iranian Registry of Clinical Trials as IRCT2015121317254N4 on 27/12/2015. The participators were overweight or obese NAFLD patients referring to the sonography department of NIOC central hospital of Tehran. This trial lasted from 8 May 2016 until 17 September 2017.

**Inclusion criteria** were NAFLD according to ultrasonography (mild to severe fatty infiltration), age 30–60 years, and 25 ≤ BMI < 35 kg/m^2^. On the other hand, **Exclusion criteria** were alcohol intake history during the past 12-months, failure to cooperate, conditions affecting the liver, secondary NAFLD, disability, uncontrolled hypertension (> 140/90 mmHg), diabetes mellitus, pregnancy or lactation, expert athlete, intake of statins, ursodeoxycholic acid, probiotics, antihypertensive, cardamom interacting drugs, and taking multivitamin-mineral and antioxidant supplements over the past 3-months, losing weight during the past 3-months, not taking more than 10% of the intervention supplement, and other criteria which have been mentioned in the published trial protocol [39, 40, 52].

This trial adhered to CONSORT guidelines and included a completed CONSORT checklist as an Additional file 1.

### Randomization and intervention

**Fig. 1:**
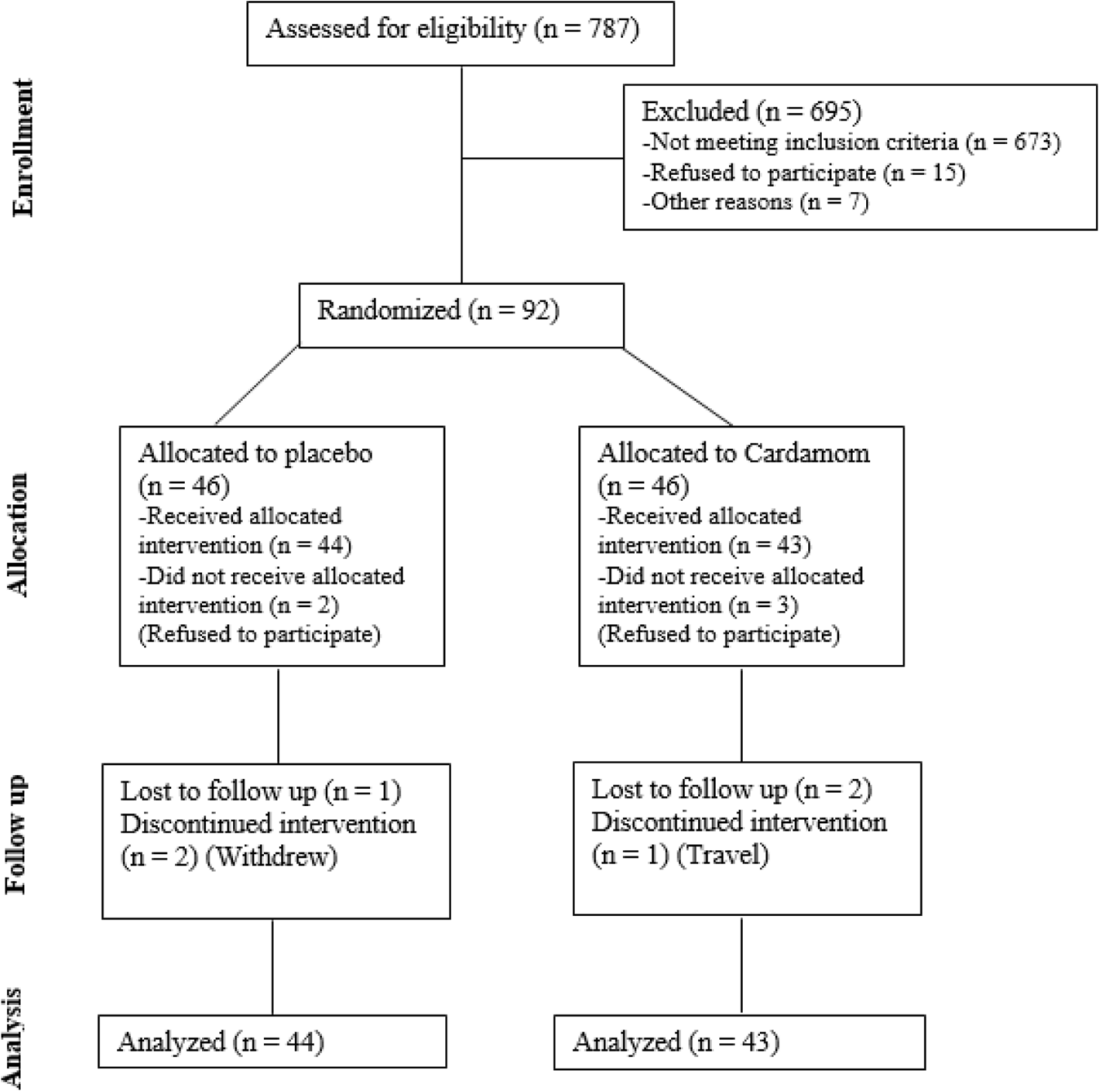
Flow chart of the study participants

According to the block randomization method, participants were divided into two equal groups by an assistant (cardamom [*n* = 43] or placebo [*n* = 44]). The stratified randomization method was utilized for matching age (30–45 and 46–60 yrs) and gender. The ratio of the two groups was 1:1. Three patients from the GC group and 2 patients from the placebo group declined to participate after randomization and before the beginning of the study (Fig. 1).

The blinding of intervention allocation was done for both the participants and investigators as A and B packages. The Samex agency of India provided GC. The GC and placebo capsules were made by the Traditional Medicine Research Center (TMRC), Iran University of Medical Sciences, Tehran, Iran. The shape, size, and color of the capsules were similar. The contents of capsules were 0.5 g of whole GC or toast powder. Before supplementation, the capsules were weekly placed alongside each other to get a similar smell. The absorbed cardamom volatile oil in the placebo capsules was very little to change health parameters. According to a previous study, the dose of supplements was determined 3 g/day [43] as 2 capsules per meal with food. However, the intake and absorption of the GC both with or without food should be investigated further. The supplements were distributed on a monthly basis and checking compliance status was also done monthly through face to face and weekly by telephone. The lifestyle advice was presented by an expert dietitian (MDM) in the hospital. The duration of the intervention was 3 months.

The GC voucher number was *Elettaria cardamomum (L.) Maton, Family: Zingiberaceae, PMP-669*. The analysis of the whole GC was performed by Medicinal Plants Institute of Shahid Beheshti University of Medical Sciences, Tehran. The contents of cardamom essential oil according to the gas chromatography-mass spectrometry (GC-MS) were 41% α-terpinyl acetate and 30% 1,8-cineole. According to high-performance liquid chromatography (HPLC) based on standard gallic acid, the total phenolic acid content was 10.53 ± 0.18 μg gallic acid equivalent/mg extract. Also, the content of total flavonoid by maceration method based on quercetin standard was 4.143 ± 1.865 μg quercetin/mg dry extract.

### -Assessments and measurements

#### General characteristics, dietary intakes, and physical activity

The NAFLD patients were identified, the eligibility criteria checked, the study details were clarified, and informed consent was obtained by the main investigator. The questionnaires including the general questionnaire, 24-h food recall (at the beginning, middle, and end), and short-form IPAQ (SF-IPAQ) questionnaire (at the beginning and end) were administered through interviewing. At the beginning of the study, lifestyle changes [41] including 5% weight loss diet [53] and enhancing physical activity as moderate-intensity aerobic at least 3 times/week for 30–45 min [54] were presented.

The dietary status was determined using gram per day of values from 24-h food recall (valid in Iran [55]) by the *Nutritionist 4* software [55, 56].

The IPAQ questionnaire provides information on physical activity that people do as part of their everyday lives. The questions are about the time when the person has been active during the last 7 days. This questionnaire addresses the activities in the workplace or as part of the homework and the garden, place to place movement, exercises, and leisure activities. It also considers all the intense activities over the past 7 days. Intense activities require a great deal of physical power and more intense breathing. The IPAQ addresses only continuous activities for at least 10 min. Its short form has 7 classified questions determining the three activity levels (1–3 or low-to-high levels). This questionnaire had been validated in Iran [57, 58].

#### Anthropometric measurements

Weight (at the beginning and end) and height (at the beginning) were determined by using a digital scale and stadiometer (*Seca® Germany, Model: 7551021994*). Body mass index (BMI) was calculated via dividing weight in kilograms by squared height in meters.

#### Sonography and blood biomarkers measurement

At the beginning and the end of the study, the liver ultrasonography was done after 12 h of fasting by one radiologist to reduce the differences of human error.

For this purpose, 10 ml blood (at the beginning and end) was taken from the peripheral vein after 12-h fasting during the night and centrifuged for 20 min (3000 *g*). Serum glucose was determined on the same day of blood withdrawal. The remaining serums were frozen and stored at − 80 °C up to the analysis.

Serum irisin was determined using the sandwich ELISA and kit as *Shanghai Crystal Day Biotech Co. Ltd®; Intra-assay CV < 8%, Inter-assay CV < 10*% by an automatic device (*Elisys Uno Human®*). Similarly, the ELISA kit for FBI was *DiaMetra® Co of Italy, DCM076–8; Intra-assay CV ≤ 5%, Inter-assay CV ≤ 10%.* Serum glucose was measured according to glucose oxidase method using Hitachi analyzer device (*q17®*) and the specific kit as *Bionik®, Liquid Stable, Glucose oxidase GOD-POD, Mono-reagent; Intra-assay CV ≤ 2.10%, Inter-assay CV ≤ 3.09%*. In addition, the serum levels of TC, TG, LDL-c, and HDL-c were measured using Hitachi analyzer device (*q17*®) and the specific kits as 1-*Bionik*®*, Liquid Stable, Enzymatic Colorimetric CHOD-POD*, 2-*Bionik*®*, Liquid Stable, GPO-POD, Mono-reagent*, 3-*Bionik*®*, Liquid Stable, Direct. Enzymatic Colorimetric*, and 4-*Bionik*®*, Liquid Stable, Direct. Enzymatic Colorimetric*, respectively. The intra- and inter-assay coefficients of variation for TC, TG, LDL-c, and HDL-c were ≤ 1.216% and ≤ 6.906%, ≤1.573% and ≤ 7.704%, ≤1.76% and ≤ 0.65%, and ≤ 0.7% and ≤ 1.5%, respectively. HOMA-IR and QUICKI indices were calculated by the following formulas:

$$ {\displaystyle \begin{array}{c} QUICKI=1/\left(\mathit{\log}\  fasting\ insulin\ \left[\mu IU/ ml\right]\right)+\mathit{\log}\  fasting glucose\ \left[ mg/ dl\right]\Big)\\ {} HOMA- IR= FBI\ \left[\mu IU/ ml\right]\times FBS\ \left[ mg/ dl\right]/405\end{array}} $$

### Sample Sizeges

The sample size was determined using the “two mean comparison formula”. According to a previous study on the effects of cardamom among type 2 diabetic patients, the mean difference of FBS between the groups was 6 mg/dl and the standard deviation was 9.6 mg/dl [51]. The type I and type II errors were considered 0.05 and 0.2, respectively. In total, 46 participants were accounted for each group (GC and placebo) while considering a 15% drop.

### Data analysis and accessibility

Data management including entry, security, coding, and storage was performed at this stage. The missing data of follow-up stage and baseline of one patient were estimated by modified-intention to treat (m-ITT) analysis and regression imputation method. The Kolmogorov-Smirnov, Chi-square, Fisher Exact, and t or Mann-Whitney tests assessed normality of continuous variables as well as categorical and continuous baseline characteristics, respectively. Two-way repeated measures analysis of variance (TWRM-ANOVA) was used to determine time effects and time by treatment interaction effects on all dependent variables. Moreover, TWRM-ANOVA was adjusted for dietary intake of vitamins E and B6. Also, 95% confidence interval (CI) and a *P*-value< 0.05 were considered for reporting the measurements. Data analysis was conducted using SPSS_16_ (statistical package for the social sciences) and STATA_11SE_ (general-purpose statistical software package by Stata Corp) software. The main investigator had access to the final dataset and the results were presented by the publication.

## Results

### Participants’ characteristics

According to Fig. 1, overall, 787 people were screened based on medical history. Specifically, 114 subjects had the eligibility criteria, of whom 15 declined and 7 could not participate. Also, 92 subjects were randomized, with 3 subjects in the GC group and 2 subjects in the placebo group refusing to participate and as such did not receive the intervention. Thus, the first visit was completed for 87 subjects (cardamom *n* = 43; placebo *n* = 44). In addition, 6 subjects could not continue the follow-up stage (for personal reasons and travel; cardamom *n* = 3; placebo *n* = 3). Further, the baseline serum sample of one subject in the placebo group was not available. Eventually, data analysis was performed for 87 subjects according to the modified-ITT analysis.

The general characteristics and physical activity level of the patients are presented in Table 1. Most of the participants had similar education, high economic and low physical activity level. Both groups used more than 95% of the prescribed supplements.

**Table 1:** General characteristics and physical activity as well as liver status of overweight or obese patients with non-alcoholic fatty liver disease (NAFLD)

General variables and physical activity		Cardamom (*n* = 43) n(%) or Mean (SD)	Placebo (*n* = 44) n(%) or Mean (SD)	*P*-value
Age (yrs)	30–45	18 (41.9)	20 (45.5)	0.7^a^
	46–60	25 (58.1)	24 (54.5)	
Gender	male	27 (62.8)	27 (61.4)	0.8^b^
Education level	up to associate degree	18 (41.9)	20 (45.5)	0.7^b^
	Bachelor and higher	25 (58.1)	24 (54.5)	
Economic level	Low/moderate (≤6 living items)	12 (27.9)	6 (13.6)	0.1^b^
	High (≥7 living items)	31 (72.1)	38 (86.4)	
Physical activity level (Baseline)		1.1 (0.3)	1.2 (0.4)	0.1^c^
Physical activity level (End)		1.3 (0.5)	1.3 (0.6)	0.6^c^
Fatty liver Degree (Baseline)	Grade 0	0 (0)	0 (0)	0.1^b^
	Grade 1	27 (62.8)	34 (77.3)	
	Grade 2	16 (37.2)	10 (22.7)	
	Grade 3	0 (0)	0 (0)	
Fatty liver Degree (End)	Grade 0	18 (41.8)	2 (4.5)	< 0.001^b^
	Grade 1	22 (51.2)	34 (77.3)	
	Grade 2	3 (7.0)	8 (18.2)	
	Grade 3	0 (0)	0 (0)	

^a^Mann-Whitney; ^b^Chi-square; ^c^t-test

**Table 2:** Comparison of baseline mean for BMI and serum irisin, glucose indices, and lipids in overweight or obese patients with non-alcoholic fatty liver disease (NAFLD)

Baseline Dependent Variables		Cardamom (*n* = 43) n(%) or Mean (SD)	Placebo (*n* = 44) n(%) or Mean (SD)	*P*-value
BMI (kg/m2)	25–29.99	16 (37.2)	17 (38.6)	0.8^b^
	30–34.99	27 (62.8)	27 (61.4)	
FBS (mg/dl)		93.3 (7.6)	91.5 (7.9)	0.3^c^
FBI (μIU/ml)		8.1 (0.6)	7.9 (0.9)	0.7^c^
HOMA-IR (score)		1.8 (0.1)	1.7 (0.2)	0.1^a^
QUICKI (score)		0.34 (0.005)	0.35 (0.008)	0.1^c^
Irisin (ng/ml)		25.3 (2.9)	25.2 (2.2)	1.0^c^
TC (mg/dl)		204.5 (25.0)	205.0 (26.7)	0.9^a^
TG (mg/dl)		170.2 (77.4)	178.0 (74.7)	0.6^a^
LDL-c (mg/dl)		125.1 (26.3)	124.4 (21.7)	0.8^a^
HDL-c (mg/dl)		44.7 (9.7)	45.3 (8.7)	0.7^a^

^a^t-test; ^b^Chi-square; ^c^Mann-Whitney; *BMI* body mass index, *HOMA-IR* homeostasis model assessment-insulin resistance, *QUICKI* quantitative insulin sensitivity check index, *FBS* fasting blood sugar, *FBI* fasting blood insulin, *TC* total cholesterol, *TG* triglyceride, *HDL-C* high-density lipoprotein cholesterol, *LDL-C* low-density lipoprotein-cholesterol

### Changes in dietary intake and blood biomarkers

The dietary intake of vitamin E in the baseline was higher in cardamom group, while the other baseline features were similar between the two groups (Tables 2 and 3).

**Table 3:** Mean of dietary intakes during the study on overweight or obese patients with non-alcoholic fatty liver disease (NAFLD)

Dietary intakes during the study	Cardamom (*n* = 43) Mean(95% CI)	Placebo (*n* = 44) Mean(95% CI)	*P*-value^a^
Energy (kcal)	2038.2 (1875.6, 2200.8)	2004.7 (1844.0, 2165.5)	0.4
Protein (g)	82.7 (74.8, 90.6)	80.6 (72.8, 88.4)	0.3
Protein (%)	16.1 (15.3, 17.0)	16.1 (15.3, 16.9)	0.6
Carbohydrate (g)	242.7 (221.5, 263.8)	245.1 (224.1, 266.0)	0.3
Carbohydrate (%)	47.5 (45.8, 49.3)	49.3 (47.5, 51.0)	0.7
Fat (g)	86.5 (78.7, 94.3)	82.2 (74.4, 89.9)	0.5
Fat (%)	38.3 (36.8, 39.8)	36.6 (35.1, 38.1)	0.8
Cholesterol (mg)	244.3 (200.6, 288.1)	221.1 (177.9, 264.3)	0.8
Saturated fat (g)	23.7 (21.2, 26.2)	22.7 (20.2, 25.1)	0.7
Monounsaturated fatty acid (g)	31.7 (28.7, 34.6)	29.8 (26.9, 32.7)	0.6
Polyunsaturated fatty acid (g)	21.9 (19.4, 24.4)	20.7 (18.2, 23.2)	0.1
Vitamin A [RAE] (μg)	372.1 (303.6, 440.6)	398.3 (330.5, 466.0)	0.3
Carotenoids (mg)	8.1 (6.61, 9.73)	8.2 (6.67, 9.75)	0.2
Vitamin C (mg)	83.1 (69.0, 97.2)	86.5 (72.6, 100.4)	0.7
Calcium (mg)	941.4 (827.2, 1055.6)	989.5 (876.6, 1102.3)	0.050
Iron (mg)	13.0 (12.0, 14.1)	12.4 (11.4, 13.5)	0.7
Vitamin D (μg)	1.0 (0.5, 1.5)	1.7 (1.2, 2.2)	0.7
Vitamin E (mg)	26.0 (23.3, 28.8)	23.5 (20.8, 26.2)	0.01
Vitamin B1 (mg)	1.54 (1.40, 1.68)	1.53 (1.40, 1.67)	0.7
Vitamin B2 (mg)	1.7 (1.5, 1.9)	1.8 (1.6, 1.9)	0.06
Vitamin B3 (mg)	23.3 (20.7, 25.9)	21.9 (19.4, 24.4)	0.4
Vitamin B6 (mg)	1.7 (1.5, 1.9)	1.6 (1.5, 1.8)	0.02
Folate (DFE) (μg)	403.2 (363.2, 443.1)	389.9 (350.4, 429.4)	0.5
Vitamin B12 (μg)	4.11 (3.55, 4.68)	4.06 (3.50, 4.62)	0.9
Vitamin K (μg)	190.4 (135.4, 245.4)	153.1 (98.8, 207.5)	0.4
Zinc (mg)	11.0 (10.0, 12.0)	11.1 (10.1, 12.1)	0.7
Selenium (μg)	95.3 (84.7, 105.9)	101.4 (90.9, 111.9)	0.7
Total fiber (g)	27.0 (24.1, 30.0)	24.7 (21.8, 27.7)	0.3

^a^Two way repeated measures-ANOVA (TWRM-ANOVA)

The dietary intake of vitamin E and B6 during the study was higher in the cardamom group (*P* < 0.05, Table 3), while the other dietary intakes were almost similar between the two groups. These significant intakes were considered as confounders in the final analysis model. Within the cardamom group, the mean difference of FBS was not significant (*P* > 0.05). On the other hand, FBI, HOMA-IR, TC, TG, and LDL-c diminished, while QUICKI, HDL-c, and irisin increased significantly (*P* < 0.05). Within the placebo group, the mean differences of FBS, TC, TG, LDL-c, and HDL-c were not significant (*P* > 0.05), but the FBI and HOMA-IR decreased and QUICKI and irisin rose significantly (*P* < 0.05) (Table 4).

**Table 4:** The changes of serum irisin, glucose indices, and lipids in overweight or obese patients with non-alcoholic fatty liver disease (NAFLD)

Variables	Supplement	Baseline Mean [SD]	End Mean [SD]	*P*-value^$^	Mean Changes (95% CI)	*P*-value^#^ (time by treatment interaction)
FBS (mg/dl)^*^	Cardamom (*n* = 43)	93.3 (7.6)	92.7 (6.7)	0.5	−0.6 (−1.0, −0.1)	0.1 0.2
	Placebo (*n* = 44)	91.5 (7.9)	92.2 (7.8)	0.09	0.7 (0.1, 1.2)	
FBI (μIU/ml)	Cardamom (*n* = 43)	8.1 (0.6)	5.4 (0.9)	< 0.001	−2.7 (−2.75, −2.64)	< 0.001 < 0.001
	Placebo (*n* = 44)	7.9 (0.9)	7.5 (1.2)	< 0.001	− 0.4 (− 0.46, − 0.33)	
HOMA-IR	Cardamom (*n* = 43)	1.8 (0.1)	1.2 (0.2)	< 0.001	−0.6 (− 0.61, − 0.59)	< 0.001 < 0.001
	Placebo (*n* = 44)	1.79 (0.2)	1.72 (0.3)	0.001	−0.07 (− 0.08, − 0.05)	
QUICKI^	Cardamom (*n* = 43)	0.347 (0.005)	0.371 (0.012)	< 0.001	0.024 (0.0246, 0.0233)	< 0.001 < 0.001
	Placebo (*n* = 44)	0.350 (0.008)	0.352 (0.010)	0.001	0.002 (0.0025, 0.0014)	
TC (mg/dl)	Cardamom (*n* = 43)	204.5 (25.0)	198.7 (25.2)	0.01	−5.8 (−7.4, −4.1)	0.1 0.2
	Placebo (*n* = 44)	205.0 (26.7)	204.7 (26.2)	0.9	−0.3 (−2.0, 1.4)	
TG (mg/dl)	Cardamom (*n* = 43)	170.2 (77.4)	134.5 (54.9)	< 0.001	−35.7 (−40.1, −31.2)	0.005 0.01
	Placebo (*n* = 44)	178.0 (74.7)	168.3 (69.0)	0.057	−9.7 (−14.3, − 5.0)	
LDL-c (mg/dl)	Cardamom (*n* = 43)	125.1 (26.3)	121.1 (24.2)	0.01	−4 (−5.6, −2.3)	0.01 0.07
	Placebo (*n* = 44)	124.4 (21.7)	126.3 (23.3)	0.3	1.9 (0.4, 3.3)	
HDL-c (mg/dl)	Cardamom (*n* = 43)	44.7 (9.7)	53.1 (10.3)	< 0.001	8.4 (7.7, 9.0)	< 0.001 < 0.001
	Placebo (*n* = 44)	45.3 (8.7)	45.7 (8.4)	0.4	0.4 (−0.1, 0.9)	
Irisin (ng/ml)^Φ^	Cardamom (*n* = 43)	25.3(2.9)	34.4 (4.3)	< 0.001	9.1 (8.8, 9.3)	< 0.001 < 0.001
	Placebo (*n* = 44)	25.2 (2.2)	26.2 (2.8)	< 0.001	1.0 (0.8, 1.1)	

*Transformed by square root; ^Cubical Inversely transformed; ΦSquare Inversely transformed; $Paired t-test; #Two way repeated measures-ANOVA (TWRM-ANOVA), top row *P*-_value_: unadjusted; bottom row *P*-_value_: adjusted for vitamins E and B6 dietary intake

*HOMA-IR* homeostasis model assessment-insulin resistance, *QUICKI* quantitative insulin sensitivity check index, *FBS* fasting blood sugar, *FBI* fasting blood insulin, *TC* total cholesterol, *TG* triglyceride, *HDL-C* high-density lipoprotein cholesterol, *LDL-C* low-density lipoprotein-cholesterol

According to the time by treatment interaction effect in the final analysis model, FBI, HOMA-IR, TG, and LDL-c declined while QUICKI, HDL-c, and irisin grew significantly among cardamom group in comparison with the placebo group (*P* < 0.05) (Table 4). In other words, GC in comparison with placebo significantly elevated irisin, HDL-c, and QUICKI and reduced FBI, HOMA-IR, TG, and LDL-c (*P* < 0.05). After adjustment for confounders, the significant changes were similar (*P* < 0.05) with an exception for LDL-c which showed a trend (*P* = 0.07) (Table 4).

After 3 months of intervention, compared with placebo, GC significantly improved the grade of fatty liver (*P* < 0.05, Table 1).

### Safety

Any side effects related to the treatment were reported, and only nausea and constipation was observed for one patient in the placebo group in one of his follow-ups.

## Discussion

This trial for the first time assessed the effects of GC on serum irisin level, glucose indices, and lipids among overweight or obese NAFLD patients. According to both unadjusted and adjusted analysis, in comparison with placebo, GC significantly augmented serum irisin, HDL-c, and QUICKI and reduced FBI, HOMA-IR, and TG levels. In addition, GC significantly lowered the grade of fatty liver. Furthermore, the decrease in LDL-c was significant in the unadjusted model and showed a trend in the adjusted model. Based on our previous findings in the other part of this trial [59], compared with placebo, GC significantly diminished serum alanine transaminase (ALT) by 127% and heightened Sirtuin 1 (Sirt1) by 40% (*P* < 0.05). In addition, the fall in BMI showed a trend among GC group in comparison with the placebo group. So, these improvements may explain the mechanism of GC effects on serum irisin, glucose indices, and lipids levels.

The results of different studies on the health effects of GC are controversial, with some of them presented further.

According to various animal studies, GC improved glycemic indices and lipids [46–48, 60–66]. In two separate clinical trials of GC effect, changes in FBS and lipids (TC, TG, LDL-c, and HDL-c) in pre-diabetic women [50] and changes in glycemic indices (FBS, insulin, and HbA1c) among type 2 diabetic patients [51], were not significant. According to the second trial among type 2 diabetic patients, GC compared with placebo significantly reduced serum TC and LDL-c and elevated HDL-c levels [51]. In addition, compared with placebo, changes in weight and BMI were not significant in both studies [50, 67]. In another study on individuals with stage 1 hypertension, GC non-significantly lowered the lipids (TC, TG, LDL-c, and VLDL-c [very-low-density lipoprotein cholesterol]) [43]. Further, Greater cardamom (*Amomum subulatum Roxb*.) in patients with ischemic heart disease significantly improved atherogenic lipids (TC, TG, LDL-c, and VLDL-c) without significant changes in HDL-c. The possible reasons for the slight contrast are the difference in the design of the study, sample size, type of patients and supplement, duration of the intervention, the level of serum glucose indices at the baseline of study. Ginger, which is another member of the Zingiberaceae family, significantly improved FBS, TC, TG, and HDL-c levels according to a meta-analysis. The reported mechanisms were related to contents of phenols, polyphenols, and flavonoids, diminished synthesis, and increased excretion of cholesterol [68].

The proposed various mechanisms of GC effect on glucose and lipid profiles include antioxidant capacity increment [63, 64], inhibition of inflammation [48], improvement of obesity, enhanced insulin activity and sensitivity (increased glycogenesis, decreased gluconeogenesis) [62], increased expression and activity of PPARγ (improved glycemic control) [46], and inhibition of cholesterol synthesis [64]. The oxidative stress may damage tissue and impair insulin secretion and glucose transmission [48]. Therefore, reducing oxidative stress can be effective in improving glucose metabolism. The hypolipidemic effect of GC may also improve plasma glucose and insulin levels and enhance insulin function [60]. The effect of flavonoids on glycemic indices is related to reduced glucose absorption and enhanced glucose tolerance [66].

Only in a cellular study on adipocytes, quercetin (as a standard flavonoid in GC) significantly amplified the expression of irisin [17]. According to the beneficial effects of irisin on glucose indices and the increase of its serum levels with GC, this new mechanism in ameliorating glycemic status is suggested through increasing irisin levels.

In a study with three levels of GC on hepatotoxicity in albino mice, a significant reduction in weight gain was observed [60]. These findings were attributed to the antioxidant capacity increment. In another study in male Wistar rats with metabolic syndrome, high levels of GC (3 g/kg body weight) augmented visceral obesity and total body fat [69]. It may be attributed to the high dose of GC.

The probable mechanism for interpreting the effects of GC on weight and BMI involves reduction of visceral fats and the absorption of foods especially fats in the gastrointestinal system and possibly increased oxidation of fats by affecting the involved enzymes [50]. In addition, these observed effects may be related to the contents of GC including phenols and flavonoids. The reported mechanisms of the effect of flavonoids on weight are the reduction of fat absorption by inhibiting pancreatic lipase [70–72], enhanced expression of PPAR-α gene and carnitine palmitoyltransferase-1 (CPT-1), and diminished gene expression of enzymes involved in fat synthesis [73]. Also, 1,8-cineole as a major component of the GC oil has significantly reduced fat mass in various studies [69].

The novelty would make this study very relevant. As the side-effects of the GC (up to 3 g/day) had not been reported previously, it may be practically feasible for patients to continue taking it in the long run. Nevertheless, the effects and durability of this intervention in the long run should be investigated. The use of the GC in some diseases especially NAFLD needs to be further studied. In addition, the emergence of obesity and, consequently, NAFLD should also be considered.

The important strengths of this study were: the earliest assessment of GC effects in overweight or obese NAFLD patients, the double-blinded stratified blocked randomization design, recruiting the participants with newly diagnosed NAFLD without any treatment, and assessing dietary intakes and physical activity status and adjusting for them. However, some limitations were the self-reporting of diet and physical activity, no liver biopsy, measuring gamma-glutamyl transferase (GGT), hemoglobin A1c (HbA1c), body composition, and bioavailability and serum levels GC or its components, and determining the durability of the intervention in the long run, and 24-h food recall which is not appropriate for determining the usual food intake.

## Conclusion

GC supplementation in overweight or obese NAFLD patients showed a significant beneficial effect on the grade of fatty liver, serum glucose indices, and lipid profiles, which may be mediated by an increase in serum Sirt1 and irisin concentration. Further trials are required to use GC in clinical practice.

## Additional file

Additional file 1: CONSORT 2010 checklist of information to include when reporting a randomized trial. (DOC 223 kb)

## Abbreviations

BMI: Body mass index
CEO: Cardamom essential oil
COX-2: Cyclooxygenase-2
ELISA: Enzyme-linked immunosorbent assay
GC: Green Cardamom
GC-MS: Gas chromatography-mass spectrometry
HPLC: High-performance liquid chromatography
hs-CRP: High-sensitivity C-reactive protein
IL-1β: Interleukin-1 beta
IL-6: Interleukin-6
iNOS: Inducible nitric oxide synthase
IPAQ: International physical activity questionnaire
ITT: Intention to treat
NAFLD: Non-alcoholic fatty liver disease
NASH: Non-alcoholic steatohepatitis
NF-κB: Nuclear factor kappa-light-chain-enhancer of activated B cells
NIOC: National Iranian Oil Company
PGC-1α: PPAR-γ coactivator-1 alpha
PPAR: Peroxisome proliferation activated receptor
ROS: Reactive oxygen species
Sirt-1: Sirtuin-1
SOD: Superoxide dismutase
TG: Triglyceride
TMRC: Traditional Medicine Research Center
TNF-α: tumor necrosis factor-alpha
TPN: Total parenteral nutrition
TWRM-ANOVA: Two way repeated measures analysis of covariance

## References

[1] Shaker M, Tabbaa A, Albeldawi M, Alkhouri N (2014). Liver transplantation for nonalcoholic fatty liver disease: new challenges and new opportunities. World J Gastroenterol: WJG.

[2] Rinella ME (2015). Nonalcoholic fatty liver disease: a systematic review. Jama..

[3] Clark JM, Diehl AM (2003). Nonalcoholic fatty liver disease: an underrecognized cause of cryptogenic cirrhosis. Jama..

[4] Adams L, Angulo P (2006). Treatment of non-alcoholic fatty liver disease. Postgrad Med J.

[5] Younossi ZM, Koenig AB, Abdelatif D, Fazel Y, Henry L, Wymer M (2016). Global epidemiology of nonalcoholic fatty liver disease—meta-analytic assessment of prevalence, incidence, and outcomes. Hepatology..

[6] Sanyal AJ (2002). AGA technical review on nonalcoholic fatty liver disease. Gastroenterology..

[7] Fabbrini E, Sullivan S, Klein S (2010). Obesity and nonalcoholic fatty liver disease: biochemical, metabolic, and clinical implications. Hepatology..

[8] Nishioji K, Sumida Y, Kamaguchi M, Mochizuki N, Kobayashi M, Nishimura T (2015). Prevalence of and risk factors for non-alcoholic fatty liver disease in a non-obese Japanese population, 2011–2012. J Gastroenterol.

[9] Musso G, Molinaro F, Paschetta E, Gambino R, Cassader M. Lipid modifiers and NASH: statins, ezetimibe, fibrates, and other agents. Non-Alcoholic Fatty Liver Disease: A Practical Guide. 2013:293–307.

[10] Gaharwar R, Trikha S, Margekar SL, Jatav OP, Ganga PD (2015). Study of clinical profile of patients of non alcoholic fatty liver disease and its association with metabolic syndrome. J Assoc Physicians India.

[11] Allocca M, Selmi C. Emerging nutritional treatments for nonalcoholic fatty liver disease. Nutr Diet Ther Liver. 2010:131–46.

[12] Nseir W, Nassar F, Assy N (2010). Soft drinks consumption and nonalcoholic fatty liver disease. World J Gastroenterol: WJG.

[13] McCullough AJ (2004). The clinical features, diagnosis and natural history of nonalcoholic fatty liver disease. Clin Liver Dis.

[14] Sahebkar A (2011). Potential efficacy of ginger as a natural supplement for nonalcoholic fatty liver disease. World J Gastroenterol.

[15] Kasper D, Fauci A, Hauser S, Longo D, Jameson J, Loscalzo J. Harrison’s principles of internal medicine, 19e. 2015.

[16] Park M-J, Kim D-I, Choi J-H, Heo Y-R, Park S-H (2015). New role of irisin in hepatocytes: the protective effect of hepatic steatosis in vitro. Cell Signal.

[17] Leiherer A, Stoemmer K, Muendlein A, Saely CH, Kinz E, Brandtner EM (2016). Quercetin impacts expression of metabolism-and obesity-associated genes in SGBS adipocytes. Nutrients..

[18] Arias-Loste MT, Ranchal I, Romero-Gómez M, Crespo J (2014). Irisin, a link among fatty liver disease, physical inactivity and insulin resistance. Int J Mol Sci.

[19] Gamas L, Matafome P, Seiça R. Irisin and myonectin regulation in the insulin resistant muscle: implications to adipose tissue: muscle crosstalk. J Diabetes Res. 2015;2015. 10.1155/2015/359159 PMC4436512 26075283

[20] Panati K, Suneetha Y, Narala V (2016). Irisin/FNDC5—an updated review. Eur Rev Med Pharmacol Sci.

[21] Sanchis-Gomar F, Perez-Quilis C (2014). The p38–PGC-1α–irisin–betatrophin axis: exploring new pathways in insulin resistance. Adipocyte..

[22] J-q C, Y-y H, Gusdon AM, Qu S (2015). Irisin: a new molecular marker and target in metabolic disorder. Lipids Health Dis.

[23] Saleh BO, Majeed MJ, Oreaby GM (2015). Irisin peptide is myokine, anti-obesity and anti-lipidemic factor. Am J Res Comput.

[24] Blüher S, Panagiotou G, Petroff D, Markert J, Wagner A, Klemm T (2014). Effects of a 1-year exercise and lifestyle intervention on irisin, adipokines, and inflammatory markers in obese children. Obesity..

[25] Dorn GW, Vega RB, Kelly DP (2015). Mitochondrial biogenesis and dynamics in the developing and diseased heart. Genes Dev.

[26] Zhang H-J, Zhang X-F, Ma Z-M, Pan L-L, Chen Z, Han H-W (2013). Irisin is inversely associated with intrahepatic triglyceride contents in obese adults. J Hepatol.

[27] Han X, Shen T, Lou H (2007). Dietary polyphenols and their biological significance. Int J Mol Sci.

[28] Brglez Mojzer E, Knez Hrnčič M, Škerget M, Knez Ž, Bren U (2016). Polyphenols: extraction methods, antioxidative action, bioavailability and anticarcinogenic effects. Molecules..

[29] Hämäläinen M, Nieminen R, Vuorela P, Heinonen M, Moilanen E (2007). Anti-inflammatory effects of flavonoids: genistein, kaempferol, quercetin, and daidzein inhibit STAT-1 and NF-kappaB activations, whereas flavone, isorhamnetin, naringenin, and pelargonidin inhibit only NF-kappaB activation along with their inhibitory effect on iNOS expression and NO production in activated macrophages. Mediators Inflamm..

[30] Kim HK, Park HR, Lee JS, Chung TS, Chung HY, Chung J (2007). Down-regulation of iNOS and TNF-α expression by kaempferol via NF-κB inactivation in aged rat gingival tissues. Biogerontology..

[31] Kim J-A, Kim D-K, Kang O-H, Choi Y-A, Park H-J, Choi S-C (2005). Inhibitory effect of luteolin on TNF-α-induced IL-8 production in human colon epithelial cells. Int Immunopharmacol.

[32] Gilmore TD (2006). Introduction to NF-κB: players, pathways, perspectives. Oncogene..

[33] Da-Silva WS, Harney JW, Kim BW, Li J, Bianco SD, Crescenzi A (2007). The small polyphenolic molecule kaempferol increases cellular energy expenditure and thyroid hormone activation. Diabetes..

[34] Davis JM, Murphy EA, Carmichael MD (2009). Effects of the dietary flavonoid quercetin upon performance and health. Curr Sport Med Rep.

[35] Dixon JB, Bhathal PS, Hughes NR, O'Brien PE (2004). Nonalcoholic fatty liver disease: improvement in liver histological analysis with weight loss. Hepatology..

[36] Shah K, Stufflebam A, Hilton TN, Sinacore DR, Klein S, Villareal DT (2009). Diet and exercise interventions reduce intrahepatic fat content and improve insulin sensitivity in obese older adults. Obesity..

[37] Rinella ME, Sanyal AJ (2016). Management of NAFLD: a stage-based approach. Nat Rev Gastroenterol Hepatol.

[38] Katan MB (2009). Weight-loss diets for the prevention and treatment of obesity. N Engl J Med.

[39] Daneshi-Maskooni M, Keshavarz SA, Mansouri S, Qorbani M, Alavian SM, Badri-Fariman M (2017). The effects of green cardamom on blood glucose indices, lipids, inflammatory factors, paraoxonase-1, sirtuin-1, and irisin in patients with nonalcoholic fatty liver disease and obesity: study protocol for a randomized controlled trial. Trials..

[40] Jazayeri-Tehrani SA, Rezayat SM, Mansouri S, Qorbani M, Alavian SM, Daneshi-Maskooni M (2017). Efficacy of nanocurcumin supplementation on insulin resistance, lipids, inflammatory factors, and nesfatin among obese patients with non-alcoholic fatty liver disease (NAFLD): a trial protocol. BMJ Open.

[41] Zelber-Sagi S, Ratziu V, Oren R (2011). Nutrition and physical activity in NAFLD: an overview of the epidemiological evidence. World J Gastroenterol: WJG.

[42] Suneetha WJ, Krishnakantha T (2005). Cardamom extract as inhibitor of human platelet aggregation. Phytother Res.

[43] Verma S, Jain V, Katewa S (2009). Blood pressure lowering, fibrinolysis enhancing and antioxidant activities of cardamom (Elettaria cardamomum). Indian J Biochem Biophys.

[44] Vijayan K, Madhusoodanan K, Radhakrishnan V, Ravindran P. Properties and end-uses of cardamom. Cardamom The genus Elettaria Ravindran PN, Madhusoodanan KJ (eds) London: Taylor & Francis. 2002:269–283.

[45] Sengupta A, Bhattacharjee S. Cardamom (*Elettaria cardamomum*) and Its Active Constituent, I, 8-cineole. Molecular Targets and Therapeutic Uses of Spices: Modern Uses for Ancient Medicine: World Scientific; 2009. p. 65–85.

[46] Bhat GN, Nayak N, Vinodraj K, Chandralekha N, Mathai P, Cherian J (2015). Comparison of the efficacy of cardamom (Elettaria cardamomum) with pioglitazone on dexamethasone-induced hepatic steatosis, dyslipidemia, and hyperglycemia in albino rats. J Adv Pharm Technol Res.

[47] El-Yamani M (2011). Cinnamon, cardamom and ginger impacts as evaluated on hyperglycemic rats. Res J Specific Educ.

[48] Rahman MM, Alam MN, Ulla A, Sumi FA, Subhan N, Khan T (2017). Cardamom powder supplementation prevents obesity, improves glucose intolerance, inflammation and oxidative stress in liver of high carbohydrate high fat diet induced obese rats. Lipids Health Dis.

[49] Yaghooblou F, Siassi F, Rahimi A, Koohdani F, Doostan F, Qorbani M (2017). The effect of cardamom supplementation on serum lipids, glycemic indices and blood pressure in overweight and obese pre-diabetic women: a randomized controlled trial. J Diabetes Metab Disord.

[50] Yaghooblou F, Siassi F, Rahimi A, Kouhdani F, Sotoudeh G (2015). The effect of cardamom supplementation on anthropometric measurements in overweight and obese Prediabetic women. Iran J Endocrinol Metab.

[51] Azimi P, Ghiasvand R, Feizi A, Hariri M, Abbasi B. Effects of cinnamon, cardamom, saffron, and ginger consumption on markers of glycemic control, lipid profile, oxidative stress, and inflammation in type 2 diabetes patients. Rev Diabet Stud: RDS. 2014;11(3):258. 10.1900/RDS.2014.11.258 PMC5397291 26177486

[52] Jazayeri-Tehrani SA, Rezayat SM, Mansouri S, Qorbani M, Alavian SM, Daneshi-Maskooni M (2018). The nanocurcumin reduces appetite in obese patients with non-alcoholic fatty liver disease (NAFLD): a double-blind randomized placebo-controlled clinical trial. Nanomedicine J.

[53] Ekhlasi G, Shidfar F, Agah S, Merat S, Hosseini KAF (2013). Effect of pomegranate juice intake on lipid profile in patients with non-alcoholic fatty liver disease. Razi J Med Sci.

[54] Musso G, Gambino R, Cassader M, Pagano G (2010). A meta-analysis of randomized trials for the treatment of nonalcoholic fatty liver disease. Hepatology..

[55] Jazayeri S, Nouri M, Pourebrahim R, Fakhrzadeh H, Larijani B (2004). Food and nutrient intakes among 20-60 aged inhabitants of Tehran University of Medical Sciences population lab region. Iran J Diab Metab.

[56] Ghaffarpour M, Houshiar-Rad A, Kianfar H (1999). The manual for household measures, cooking yields factors and edible portion of foods. Tehran: Nashre Olume Keshavarzy.

[57] Baghiani-Moghaddam MH, Bakhtari-Aghdam F, Asghari-Jafarabadi M, Allahverdipour H, Dabagh-Nikookheslat S, Nourizadeh R. Comparing the Results of Pedometer-Based Data and International Physical Activity Questionnaire (IPAQ). J Health Syst Res. 2013;9(6):605-12. [in Persian].

[58] Vafainajar A, Vahedian-Shahroodi M, Tehrani H, Dogonchi M, Lael-Monfared E (2015). The effectiveness of physical activity training on depersonalization and lack of accomplishment of employees.

[59] Daneshi-Maskooni M, Keshavarz SA, Qorbani M, Mansouri S, Alavian SM, Badri-Fariman M, Jazayeri-Tehrani SA, Sotoudeh G (2018). Green cardamom increases Sirtuin-1 and reduces inflammation in overweight or obese patients with non-alcoholic fatty liver disease: a double-blind randomized placebo-controlled clinical trial. Nutr Metab.

[60] Aboelnaga S (2015). Effect of some levels of cardamom, clove and anise on hepatotoxicity in rats caused by CCL4. World Appl Sci J.

[61] Aboubakr M, Abdelazem AM (2016). Hepatoprotective effect of aqueous extract cardamom against gentamicin induced hepatic damage in rats. Int J Basic Appl Sci.

[62] Alshammari GM (2017). Combined effect of Arabian coffee, cardamom and cloves on obesity associated insulin resistance in high-fat diet (HFD)-induced C57BL/6J mice. Res J Biotechnol.

[63] Asimi O, Sahu N. Effect of antioxidant rich spices, clove and cardamom extracts on the metabolic enzyme activity of Labeo rohita. J Fisheries Livest Prod. 2016:1–6.

[64] Darwish M, Abd EA (2013). Role of cardamom (Elettaria cardamomum) in ameliorating radiation induced oxidative stress in rats. Arab J Nuclear Sci Appl.

[65] Sadeek EA, El-Razek FHA (2010). The chemo-protective effect of turmeric, chili, cloves and cardamom on correcting iron overload-induced liver injury, oxidative stress and serum lipid profile in rat models. J Am Sci.

[66] Winarsi H, Sasongko N, Purwanto A, Nuraeni I (2014). Effect of cardamom leaves extract as antidiabetic, weight lost and hypocholesterolemic to alloxan-induced Sprague Dawley diabetic rats. Int Food Res J.

[67] Azimi P, Ghiasvand R, Feizi A, Hosseinzadeh J, Bahreynian M, Hariri M (2016). Effect of cinnamon, cardamom, saffron and ginger consumption on blood pressure and a marker of endothelial function in patients with type 2 diabetes mellitus: a randomized controlled clinical trial. Blood Press.

[68] Jafarnejad S, Keshavarz SA, Mahbubi S, Saremi S, Arab A, Abbasi S (2017). Effect of ginger (Zingiber officinale) on blood glucose and lipid concentrations in diabetic and hyperlipidemic subjects: a meta-analysis of randomized controlled trials. J Funct Foods.

[69] Bhaswant M, Poudyal H, Mathai ML, Ward LC, Mouatt P, Brown L (2015). Green and black cardamom in a diet-induced rat model of metabolic syndrome. Nutrients..

[70] Kawaguchi K, Mizuno T, Aida K, Uchino K (1997). Hesperidin as an inhibitor of lipases from porcine pancreas and pseudomonas. Biosci Biotechnol Biochem.

[71] Martins F, Noso TM, Porto VB, Curiel A, Gambero A, Bastos DH (2010). Maté tea inhibits in vitro pancreatic lipase activity and has Hypolipidemic effect on high-fat diet-induced obese mice. Obesity..

[72] Sbarra V, Ristorcelli E, Le Petit-Thévenin J, Teissedre P-L, Lombardo D, Vérine A. In vitro polyphenol effects on activity, expression and secretion of pancreatic bile salt-dependent lipase. Biochimica et Biophysica Acta (BBA)-molecular and cell biology of Lipids. 2005;1736(1):67–76. 10.1016/j.bbalip.2005.06.009 16099206

[73] Galleano M, Calabro V, Prince PD, Litterio MC, Piotrkowski B, Vazquez-Prieto MA (2012). Flavonoids and metabolic syndrome. Ann N Y Acad Sci.

