# Supplementary material for: Green cardamom supplementation improves serum irisin, glucose indices, and lipid profiles in overweight or obese non-alcoholic fatty liver disease patients: a double-blind randomized placebo-controlled clinical trial
Source: BMC Complement Altern Med. 2019 Mar 12;19:59. doi: 10.1186/s12906-019-2465-0 (PMC6419418; doi:10.1186/s12906-019-2465-0)
Supplement: Supplementary file 1 — CONSORT 2010 checklist of information to include when reporting a randomized trial. (DOC 223 kb) [file 12906_2019_2465_MOESM1_ESM.doc]

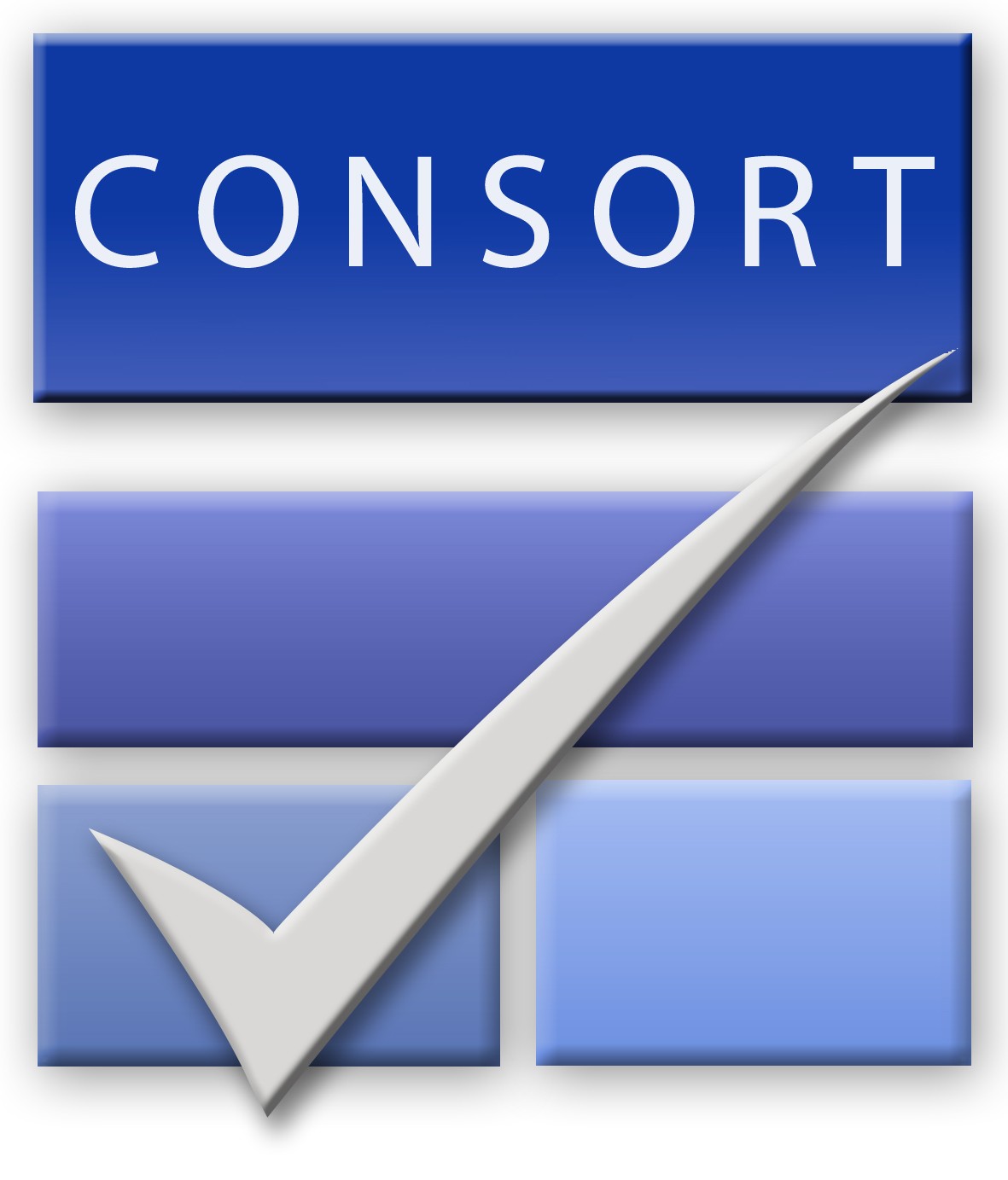
CONSORT 2010 checklist of information to include when reporting a randomized trial*

| Section/Topic | Item No | Checklist item | Reported on page No |
| --- | --- | --- | --- |
| Title and abstract | | | |
|  | 1a | Identification as a randomized trial in the title | The title, Page 1 |
|  | 1b | Structured summary of trial design, methods, results, and conclusions (for specific guidance see CONSORT for abstracts) | Abstract, Page 2 |
| Introduction | | | |
| Background and objectives | 2a | Scientific background and explanation of the rationale | Background, Paragraphs 1-6 |
|  | 2b | Specific objectives or hypotheses | Background, Paragraph 7 |
| Methods | | | |
| Trial design | 3a | Description of trial design (such as parallel, factorial) including allocation ratio | Methods, Page 5, Paragraphs 1 & 4 |
|  | 3b | Important changes to methods after trial commencement (such as eligibility criteria), with reasons | N/A |
| Participants | 4a | Eligibility criteria for participants | Methods, Page 5, Paragraph 2 |
|  | 4b | Settings and locations where the data were collected | Methods, Pages 5 & 6, Paragraphs 1 & 5 |
| Interventions | 5 | The interventions for each group with sufficient details to allow replication, including how and when they were actually administered | Methods, Page 6, Paragraphs 5 & 6 |
| Outcomes | 6a | Completely defined pre-specified primary and secondary outcome measures, including how and when they were assessed | Methods, Pages 6-8, Paragraphs 7-13 |
|  | 6b | Any changes to trial outcomes after the trial commenced, with reasons | N/A |
| Sample size | 7a | How sample size was determined | Methods, Pages 8 & 9, Sample Size |
|  | 7b | When applicable, explanation of any interim analyses and stopping guidelines | N/A |
| Randomisation: |  |  |  |
| Sequence generation | 8a | Method used to generate the random allocation sequence | Methods, Pages 5 & 6, Paragraphs 4 & 5 |
|  | 8b | Type of randomization; details of any restriction (such as blocking and block size) | Methods, Pages 5 & 6, Paragraphs 4 & 5 |
| Allocation concealment mechanism | 9 | The mechanism used to implement the random allocation sequence (such as sequentially numbered containers), describing any steps taken to conceal the sequence until interventions were assigned | They exist but not explained |
| Implementation | 10 | Who generated the random allocation sequence, who enrolled participants, and who assigned participants to interventions | They exist but not explained |
| Blinding | 11a | If done, who was blinded after assignment to interventions (for example, participants, care providers, those assessing outcomes) and how | Methods, Page 6, Paragraphs 5 |
|  | 11b | If relevant, description of the similarity of interventions | Methods, Page 6, Paragraph 5 |
| Statistical methods | 12a | Statistical methods used to compare groups for primary and secondary outcomes | Methods, Page 9, Data Analysis |
|  | 12b | Methods for additional analyses, such as subgroup analyses and adjusted analyses | Methods, Page 9, Data Analysis |
| Results | | | |
| Participant flow (a diagram is strongly recommended) | 13a | For each group, the numbers of participants who were randomly assigned received intended treatment and were analyzed for the primary outcome | Results, Paragraph 1 and Figure 1 |
|  | 13b | For each group, losses, and exclusions after randomization, together with reasons | Results, Paragraph 1 and Figure 1 |
| Recruitment | 14a | Dates defining the periods of recruitment and follow-up | Methods, Study Design, Paragraph 1, Randomization, Paragraph 2 |
|  | 14b | Why the trial ended or was stopped | N/A |
| Baseline data | 15 | A table showing baseline demographic and clinical characteristics for each group | Results, Paragraphs 1-3 and Tables 1-3 |
| Numbers analyzed | 16 | For each group, number of participants (denominator) included in each analysis and whether the analysis was by original assigned groups | Results, Paragraph 1 and Figure 1 |
| Outcomes and estimation | 17a | For each primary and secondary outcome, results for each group, and the estimated effect size and its precision (such as a 95% confidence interval) | Results, Paragraphs 4-6 and Tables 1 & 3 & 4 |
|  | 17b | For binary outcomes, the presentation of both absolute and relative effect sizes is recommended | Results, Paragraphs 4-6 and Table 4 |
| Ancillary analyses | 18 | Results of any other analyses performed, including subgroup analyses and adjusted analyses, distinguishing pre-specified from exploratory | Results, Paragraphs 5 & 6 and Table 4 |
| Harms | 19 | All important harms or unintended effects in each group (for specific guidance see CONSORT for harms) | Results, Safety |
| Discussion | | | |
| Limitations | 20 | Trial limitations, addressing sources of potential bias, imprecision, and, if relevant, a multiplicity of analyses | Discussion, The last paragraph |
| Generalisability | 21 | Generalisability (external validity, applicability) of the trial findings | Discussion, Paragraphs 3-7 |
| Interpretation | 22 | Interpretation consistent with results, balancing benefits and harms, and considering other relevant evidence | Discussion, Paragraphs 3-7 |
| Other information | | |  |
| Registration | 23 | Registration number and name of trial registry | Below of the Abstract, Methods, Paragraph 1 |
| Protocol | 24 | Where the full trial protocol can be accessed, if available | Reference 39, Page 18 (DOI: 10.1186/s13063-017-1979-3) |
| Funding | 25 | Sources of funding and other support (such as the supply of drugs), the role of funders | Declaration, Funding |

*We strongly recommend reading this statement in conjunction with the CONSORT 2010 Explanation and Elaboration for important clarifications on all the items. If relevant, we also recommend reading CONSORT extensions for cluster randomized trials, non-inferiority and equivalence trials, non-pharmacological treatments, herbal interventions, and pragmatic trials. Additional extensions are forthcoming: for those and for up to date references relevant to this checklist, see [www.consort-statement.org](http://www.consort-statement.org/).
